# Workplace Violence: Impact on the Commitment and Involvement of Nurses at Work

**DOI:** 10.1155/2023/9987092

**Published:** 2023-06-27

**Authors:** Yolanda Navarro-Abal, José Antonio Climent-Rodríguez, Rosa María Vaca-Acosta, Javier Fagundo-Rivera, Juan Gómez-Salgado, Juan Jesús García-Iglesias

**Affiliations:** ^1^Department of Social, Evolutionary and Educational Psychology, Faculty of Labour Sciences, University of Huelva, Huelva, Spain; ^2^Department of Business, Management and Marketing, Faculty of Labour Sciences, University of Huelva, Huelva, Spain; ^3^Red Cross Nursing University Centre, University of Seville, Seville, Spain; ^4^Department of Sociology, Social Work and Public Health, Faculty of Labour Sciences, University of Huelva, Huelva, Spain; ^5^Safety and Health Postgraduate Programme, University Espíritu Santo, Guayaquil, Ecuador

## Abstract

**Objective:**

To analyse the relationship between workplace violence and work engagement in nursing professionals.

**Background:**

Violence in the workplace has become a phenomenon of growing concern. Nursing professionals are one of the most exposed groups to this type of violence.

**Method:**

A descriptive cross-sectional study was carried out on a national sample of Spanish nurses by means of convenience, non-probabilistic sampling. A questionnaire was administered that collected sociodemographic variables, the Utrecht Work Engagement Scale (UWES-9), and an “ad hoc” scale on violence in the workplace.

**Results:**

1648 active nursing professionals participated in this study. The participants obtained higher scores in the “dedication” variable. It was observed that 42.17% had personally suffered some type of aggression or violence at work. Verbal aggression was the most frequent type of reported violence. Nurses who experience violence, threat, and psychological harassment showed lower levels of work engagement than those who did not. In the case of those who experience sexual harassment and those who do not, the values for the UWES-9 were similar.

**Conclusions:**

Having personally experienced violence, threats or psychological harassment can negatively affect nurses' work engagement, the most common acts of violence being verbal and often perpetrated by family members and patients. *Implications for Nursing Management*. Adequate tools are not still being used to ensure that the assaulted professionals are adequately assisted by their institutions.

## 1. Background

Although there are numerous definitions of workplace violence, the International Labour Organisation (ILO) defines it as any action, incident, or behaviour that departs from reasonable conduct in which a person is assaulted, threatened, harmed, and injured in the course of, or as a direct result of, his or her work. Internal workplace violence takes place between workers, including managers and supervisors, while external violence takes place between workers (and managers and supervisors) and any other person present at the workplace [1].

In addition to the previous definition, there are several classifications of workplace violence. One of the most recognised in the scientific context is the one established by the California Division of Occupational Safety and Health (Cal/OSHA) [2]. It was originally proposed as a way of systematising the manifestations of violence involving risk to life or physical harm. Subsequently, more subtle forms of violence were categorised using the person who carries out the violent behaviour as a differentiating criterion. Thus, three types of violence can be differentiated: type I, caused by external agents with no legitimate link to the victim; type II, caused by clients, customers, patients, and consumers; and finally, type III, caused by colleagues, subordinates, or managers.

Workplace violence is internationally recognised as an occupational hazard for health professionals and has become a major global concern in recent years [3] due to an increase in situations of violence and aggressiveness in the workplace. As indicated by the National Institute for Safety, Health, and Welfare at Work in 2018, there has been an increase in psychosocial risks as regards workplace violence understood as harassment, threats, or aggression [4]. It is clear that workplace violence is a major occupational hazard, affecting both the health and safety of health workers [5].

This situation led to the ratification of the ILO Convention No. 190 (C190) [6]. It is the first international treaty to recognise the right of everyone to a world free from violence and harassment, including violence and harassment based on sex or gender. The Convention was ratified in June 2019 by the ILO's International Labour Conference and entered into force on 25 June 2021. The treaty must put in place the necessary laws and policy measures to prevent and address violence and harassment in the world of work [6]. Therefore, there is a growing need to identify both individual and contextual variables that influence the development of workplace violence. Only in this way will it be possible to promote organisational strategies to prevent and mitigate possible cases of violence.

More specifically, due to the frequency with which it is detected, special mention should be made of workplace violence among health professionals, such as nurses. There exists global concern about this issue, as this type of violence is internationally recognised as an occupational hazard for health professionals [7].

Given the difficulty in eradicating the occurrence of these situations due to the influence of multiple factors, it is necessary to cushion this negative impact by following an approach towards health and well-being at work, with the aim of trying to promote an optimal psychosocial functioning of workers [8, 9]. In this context, the functional or dysfunctional links between workers and their work activity have an impact on their health and well-being, hence the importance of analysing the variables that may act as modulators of occupational health, such as engagement [10].

Salanova and Schaufeli [11] identified three psychological conditions that engaged people manifest: (1) satisfaction: the work itself is meaningful and challenging for the person; (2) safety: the workplace is reliable, safe, and predictable, generating a positive work climate for the person; and (3) availability: the physical and psychological resources that people have are necessary and available to improve the work role performed.

Engagement is currently analysed from two different perspectives, i.e., the role theory and occupational health. Both coincide in that this process integrates three components: vigour, absorption, and dedication [12]. Vigour (behavioural-energetic component) is characterised by high levels of energy and mental activation at work, willingness and readiness to invest effort, and persistence, even in the face of difficulties (as opposed to emotional exhaustion); absorption (cognitive component), on the other hand, is characterised by a state of concentration, a feeling that time passes quickly and one finds it difficult to detach oneself from work (as opposed to self-fulfilment/academic effectiveness); finally, dedication (emotional component) implies involvement, enthusiasm, pride, and inspiration in one's work and is characterised by a feeling of importance and challenge (as opposed to cynicism). Levels of work engagement among healthcare professionals tend to range from intermediate to high [13, 14]. In the case of a sample of Spanish nurses during the COVID-19 pandemic, the mean of the total value of work engagement was 4.6 points (SD = 1.35) [15], as opposed to the high level of work engagement found in samples of nurses from other countries such as the Kingdom of Saudi Arabia (*M* = 5.47, SD = 0.908) [16].

In this sense, work engagement, from the perspective of positive psychology, becomes a key element in organisations when disruptive situations occur, such as, for example, aggressions, which could reduce the individual component and, in turn, have an impact on the organisational component [17]. It has also been observed that work engagement could be associated with patient satisfaction, the frequency of errors, and the quality of the services provided, as well as with the work climate [18, 19].

Against this background, the aim of the study was to analyse workplace violence and its relationship to engagement in nursing professionals. In this sense, it is hypothesised that experiencing violence in the workplace reduces levels of work engagement in nurses.

## 2. Methods

### 2.1. Study Design

A descriptive cross-sectional study was carried out using questionnaires.

### 2.2. Population and Sample

#### 2.2.1. Scope and Study Population

The study population consisted of 330,745 nurses who were registered in Spain on the year 2021 [20].

#### 2.2.2. Sample

*Convenience, Nonprobabilistic Sampling*. The estimated sample size was calculated by considering a confidence level of 95%, a precision level of 2.5%, and an expected proportion of losses of 0.15 out of the total accessible population.

According to these characteristics, the total sample selected was 1808 people, of which 160 subjects were finally eliminated as they did not fully complete the questionnaire, which left a final sample of 1648 subjects. The inclusion criteria were (1) nurses who carry out care, teaching, management, and/or research work in their profession; (2) residents in the national territory (Spain); and (3) those who have accepted the informed consent.

### 2.3. Variables and Instruments

#### 2.3.1. Variables

Different sociodemographic variables (age, sex, and place of residence) and other variables of interest related to the objective of the study (professional category, position held, type of contract, length of service in the institution, and work shifts) were collected.

#### 2.3.2. Instruments

*(1) Utrecht Work Engagement Scale (UWES-9*) [21]. This scale assesses engagement through 9 items with a Likert-type response format and consists of three dimensions: vigour, dedication, and absorption. It offers three partial scores and a total score, establishing six values in a range between zero and six points. For its calculation, these values were recoded according to the following classification: high, from the range between the maximum value and the 75^th^ percentile (max ≤ UWES-9 < 75^th^ percentile); intermediate, from the 75^th^ to the 25^th^ percentile (75^th^percentile ≤ UWES-9 < 25^th^ percentile); and low, values below or equal to the 25^th^ percentile (25^th^ percentile ≤ UWES-9 ≤ minimum). Internal consistency on all three dimensions offered Cronbach values that were equal to or exceeded the critical value of 0.70 [21], more specifically 0.821.

#### 2.3.3. Workplace Violence Instrument

An “ad hoc” evaluation instrument was designed to assess the signs and indicators of workplace violence. It consisted of eight closed-response items, three of which were single-choice and five of which were multiple-choice. The questions were as follows. Have you ever suffered or witnessed violence in your workplace? What kind of relationship did victim and aggressor have? What kind of aggression did you suffer? Who was the aggressor(s)? Have you suffered any threats? From whom did you suffer the threat? Have you suffered sexual harassment in your workplace? And, have you suffered psychological harassment in your workplace? To ensure the reliability of the instrument, an inter-judge assessment was carried out. All the judges were professionals involved in management processes, occupational risk prevention, and/or coexistence commissions. More specifically, there were 5 nurses (2 hospital nurses, 2 health centre nurses, and 1 nurse from a social health centre), 1 person from the hospital's occupational risk prevention service, 1 hospital nursing supervisor (intermediate position), 2 health psychologists, and 3 volunteers, all belonging to the School of Patients of Andalusia (Granada, Spain).

### 2.4. Procedure

The sample was collected via a web link to Google Forms©. The evaluation instrument was distributed by the General Nursing Council of Spain and the Nursing Associations of each Spanish province. In Spain, there is only one General Council of Nursing (located in Madrid) (level I), and this in turn coordinates and/or governs the 17 Autonomous Councils (level II) and 52 Provincial Colleges (level III). Both level II and level III are subordinated to level I. For this purpose, they made use of the official contact they had registered as a means of notifying information and disseminated the invitation to participate through their official websites and social networks, as well as via WhatsApp, Twitter, and LinkedIn. Responses were sought from all Provincial Nursing Councils in the country. Collection was carried out in the period between January 2019 and January 2020. Two attempts were made, an initial one in January 2019 and another after 6 months, to collect all possible responses.

### 2.5. Statistical Analysis

Descriptive statistics are presented as percentages and frequencies. The Kolmogorov–Smirnov test was used to determine whether the data showed a normal distribution. On the other hand, the Kruskal–Wallis test was used to verify differences in the groups analysed with respect to the assessment of the different dimensions. Furthermore, the Mann–Whitney *U* test, with Bonferroni correction, was used to analyse which subgroups differed from each other. Finally, the results of the CHAID algorithm were analysed [22, 23], which allowed assessing the association between the variables that make up the “engagement” dimension and workplace violence. The SPSS Statistics 26.0 software was used in the study.

### 2.6. Ethical Considerations

The participants took part in the study on a voluntary basis and accepted the principles of the informed consent in which the purpose of the research was explained. Their anonymity and confidentiality were respected at all times.

The study was conducted in accordance with the requirements of the Ethical Principles for Medical Research Involving Human Subjects contained in the latest version of the Declaration of Helsinki (Fortaleza Amendment, Brazil, October 2013). It was also approved by the Ethics Committee of the General Nursing Council (Spain) in April 2018.

Moreover, the data obtained during the study were processed in accordance with Organic Law 3/2018, of 5 December, on Personal Data Protection and Guarantee of Digital Rights.

## 3. Results

The sample included 86.2% (*n* = 1420) of women and 13.8% (*n* = 228) of men. The age range was between 40 and 45 years, with women having *M* = 41.5 (SD = 10.7) and men having *M* = 43.1 (SD = 11.6). These results coincide with the statistical data on registered nursing professionals provided in [20].

### 3.1. Engagement Analysis (UWES-9)

In relation to the “engagement” variable, Table 1 shows the data from the descriptive analysis. It is observed that the participants had *M* = 4.24 (SD = 1.3), with the “dedication” dimension obtaining the highest score (*M* = 4.38; SD = 1.4) and the “vigour” dimension obtaining the lowest (*M* = 4.06, SD = 1.36).

The data showed a moderate asymmetric behaviour towards the left for the total score and for its three dimensions of the UWES-9. The kurtosis of the dedication, absorption, and total score was leptokurtic, yet the kurtosis of the “vigour” dimension was platykurtic. In none of the cases the scores showed normality.

### 3.2. Descriptive Analysis of Workplace Violence

As regards the indicators or signs of violence or aggression at work, as shown in Table 2, 42.17% (*N* = 695) had personally suffered some type of aggression or violence in the workplace, compared to 57.83% (*N* = 953) who stated the opposite.

As for the type of aggression (Table 3), it can be seen that the most frequent type of aggression was verbal, which accounted for 54.73% (*N* = 902) of the sample, compared to physical aggression, which accounted for 2% (*N* = 33).

In relation to the persons who inflict some type of aggression or act of violence (Table 4), the data indicate that 16.99% (*N* = 280) stated it was the patients and 16.26% (*N* = 268) stated it was the patients' relatives. Likewise, 13.35% (*N* = 220) stated that it was their own colleagues who were responsible for these acts.

With regard to threats, 41.20% (*N* = 679) of the participants stated that they had been threatened. Relatives (13.23%, *N* = 218) and patients (8.37%, *N* = 138) were the main perpetrators. However, aggressions were also observed to come from different sources, with 14.81% of the nurses stating that the perpetrators were family members and patients; 4% were colleagues and patients; and, finally, 4.61% stated that they had been assaulted by colleagues and also by family members.

On the other hand, 7.6% (*N* = 126) reported having suffered sexual harassment at work, and 38.90% (*N* = 641) reported having suffered psychological harassment at work (Table 5).

### 3.3. Correlational Analysis of Workplace Violence and Engagement

As shown in Table 6, it can be seen that having personally experienced violence, threats, or psychological harassment showed significant differences for all dimensions of the UWES-9 scale and for the total score. However, there were no significant differences in the case of having suffered sexual harassment.

### 3.4. CHAID Algorithm

**Figure 1:**
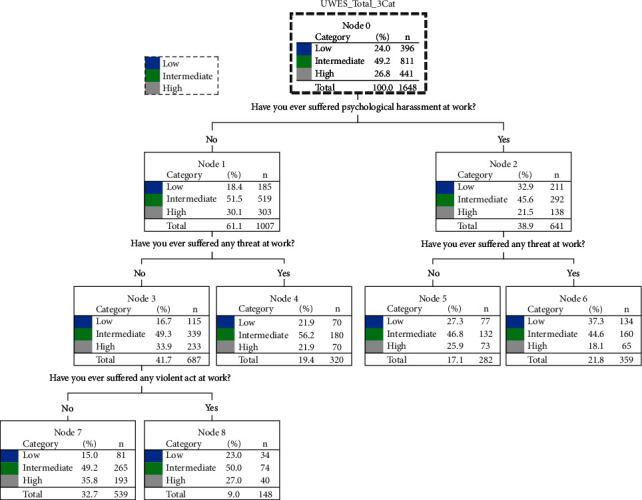
CHAID algorithm.

Figure 1 allows for the determination of the most significant variables according to the total scores of the UWES-9. At a first level, the people who reported having suffered psychological harassment at work are displayed. Although the category that shows a greater number of cases includes the mean of having suffered psychological harassment or not, the percentages of low level of engagement decrease and, conversely, in the case of not having suffered psychological harassment, percentages of high level of engagement increase. On a second level, suffering a threat is presented as the most significant variable in both cases, with percentages varying as in the previous nodes. Finally, the fact of not having suffered psychological harassment or threats is mediated having personally suffered a violent situation at work.

On the other hand, the final nodes show that people who did not suffer psychological harassment, threats, or violence at work (35.8%) had a high level of engagement compared to 15% of the participants who showed low levels. At the other end of the scale, of those who had personally experienced psychological harassment, threats, and violence, 18.1% had a high level of engagement and 37.3% had a low level of engagement. The percentages ranged between 27.0% (high engagement) and 23.0% (low engagement) among those who only suffered some form of violence.

## 4. Discussion

Workplace violence is a clear threat to both the physical and mental health of workers. It is therefore a very relevant aspect of occupational health that requires the utmost attention, as this factor can act as a cause of or trigger physical and mental illnesses.

The analysis of the classification tree (Figure 1) shows that aggressions, especially those of a psychological nature, have a relevant influence on work engagement, since 35.8% of participants who did not suffer psychological harassment nor violent acts obtained a high score in the “engagement” variable, while in the case of having suffered these situations, only 18.1% obtained a high score. This study shows that the level of engagement fluctuates according to whether the participants have been victims of psychological harassment. Thus, a lower score in psychological harassment coincides with a higher score in engagement, and vice versa, which shows that suffering psychological harassment influences the level of engagement. It can be observed that having personally experienced violence, threats, or psychological harassment showed significant differences for all dimensions of the UWES-9 scale and for the total score, but no significant differences were found in the case of having experienced sexual harassment. These results support the initial hypothesis except for sexual violence. On the other hand, and in relation to the other variable of interest in this study, work engagement, the values measured through the UWES-9 were moderately high in the sample (4.24, maximum 6).

According to Innstrand et al. [24], there is a relationship between engagement and a lower likelihood of developing anxiety and depression in response to work stressors. However, anxiety and depression are also predictors of reduced ability to stay engaged in the face of equal job demands, resource constraints, or stressors such as harassment [25]. It is necessary to point out that, at the time the present research was developed, no studies had been found that assessed the relationship between the variables raised in the study at hand.

There are several studies that warn of the high incidence of workplace violence among public administration workers who are considered to be a risk group [26]. As an example of the most vulnerable groups, the teaching staff sector is highlighted; violence against teachers by students is a well-known and common reality [27, 28]. Moreover, these data have been obtained in different geographical settings, so it can be inferred that this is a widely known issue of concern [29]. In this sense, nursing is the most affected professional group in the health sector [30], and nurses are at a higher risk of suffering violence from users [31] and also from their own work colleagues [32], most frequently threats and verbal violence, although physical aggression has increased in recent years [33], with men being the most at risk. On the other hand, in relation to sexual aggression and sexual harassment, it is women who are most at risk [34].

In addition, nurses are often the first to experience some form of workplace violence, possibly because of their closer proximity to patients and families, although it is medical professionals who report a higher prevalence of physical violence [35]. Whether there is an under-reporting of non-physical aggression is under discussion, but it is a possible factor to consider in these scenarios.

Thus, it is necessary to specifically approach this problem in order to understand both its magnitude and its influence on people's health and also, if possible, to identify the most vulnerable and potentially risk groups. In this sense, special emphasis should be placed on the nursing profession, which is mainly composed of female professionals. The present study sample belongs to a mainly female population, so the sex bias in its composition, with more than 80% women (86.17%), could be relative. This fact was considered, and no additional analyses were performed on sex differences in this study. However, this proportion is in line with that of the general nursing population. It is important to highlight that some authors, such as Davidson et al. [36], noted that suicide rates among female nurses are significantly higher (10 per 100,000) than those in the general female population (7 per 100,000). They also stated that suicide rates for nurses (33 per 100,000) were higher than those for the general male population (27 per 100,000) in the same period. On the other hand, they indicated other relevant consequences such as consumption of legal and illegal substances, mental health problems, chronic pain, absenteeism, or dismissal from work. Similarly, in relation to the greater vulnerability of women, international studies have described important sex inequalities in the field of mental health, as it is women who report and are more frequently diagnosed with mental health problems than men [37].

### 4.1. Limitations

As stated, the proportion of female nurses in this study (80% vs. 20% of men) is in line with that of the general nursing population. However, these percentages led the authors to consider not continuing with analyses other than descriptive ones on the sex variable. In addition, there could be a possible selection bias regarding the study population, as it is subjected to the degree of interest of the professionals in participating, in addition to the use of self-administered questionnaires; given this fact, researchers must rely on the veracity of the data provided by the participants. Another limitation may be related to the type of sampling used as, being non-probabilistic, it allows for an estimation of the results, but the sample is not representative.

## 5. Conclusions

In general terms, 42.17% of the sample had personally suffered some type of aggression or violence in the workplace, and the most frequent type of aggression was verbal. 41.20% of the participants stated that they had been threatened. In relation to the perpetrators of violence and threats, patients and patients' relatives were the most commonly reported groups.

It can be observed that having personally experienced violence, threats, or psychological harassment showed significant differences in all dimensions of work engagement, but not in the case of having experienced sexual harassment. These results support the initial hypothesis of this study that experiencing workplace violence reduces levels of work engagement in nurses, with the exception of sexual violence.

Finally, further research should explore the relationship between work engagement and workplace violence with respect to the sex variable. These analyses may be interesting in order to identify the main differences regarding the forms of psychological harassment and sexual harassment and also to visualise the main perpetrators of violence. In relation to sexual harassment, addressing this topic in further studies that focus specifically on this issue is considered of vital importance.

## 6. Implications for Nursing Management

There is a relationship between having suffered any form of aggression and the degree of engagement of the professionals. This aspect is of key relevance as it highlights the possibility that adequate tools are not being used to ensure that the assaulted professionals are adequately assisted by their institutions, and therefore their degree of engagement decreases. In this sense, the responsibility falls on these institutions to confront and offer solutions that contribute to alleviating the problem by establishing preventive policies and effective actions, for which they must count on the opinion and participation of nursing professionals.

## Figures and Tables

**Table 1:** Descriptive and normality study for dimension scores and UWES-9 total score, overall and by sex.

	UWES	M	SD	Asymmetry	Kurtosis	Min	Quartiles			Max	KS
							25	50	75		
Total	*V*	4.06	1.36	−0.66	−0.27	0	3.00	4.33	5.00	6	0.134^*∗∗*^
	*D*	4.38	1.44	−0.98	0.36	0	3.67	4.67	5.33	6	0.141^*∗∗*^
	*A*	4.27	1.42	−0.97	0.37	0	3.67	4.67	5.33	6	0.140^*∗∗*^
	Total	4.24	1.30	−0.83	0.08	0	3.44	4.56	5.22	6	0.102^*∗∗*^

Females	*V*	4.05	1.35	−0.63	−0.33	0	3.00	4.33	5.00	6	0.132^*∗∗*^
	*D*	4.39	1.41	−0.94	0.28	0	3.67	4.67	5.58	6	0.134^*∗∗*^
	*A*	4.28	1.41	−0.97	0.41	0	3.67	4.67	5.33	6	0.138^*∗∗*^
	Total	4.24	1.28	−0.80	0.01	0	3.44	4.56	5.22	6	0.100^*∗∗*^

Males	*V*	4.08	1.42	−0.82	0.05	0	3.33	4.33	5.00	6	0.146^*∗∗*^
	*D*	4.32	1.58	−1.14	0.53	0	3.67	4.67	5.33	6	0.182^*∗∗*^
	*A*	4.20	1.49	−0.96	0.19	0	3.67	4.67	5.33	6	0.150^*∗∗*^
	Total	4.20	1.41	−0.95	−0.28	0	3.44	4.56	5.22	6	0.125^*∗∗*^

*V* = vigour; *D* = dedication; *A* = absorption; KS = Kolmogorov–Smirnov test. ^*∗∗*^*p* < 0.001.

**Table 2:** Violence at the workplace.

Have you ever suffered or witnessed violence in your workplace?	Total		Females		Males	
	Cases	%	Cases	%	Cases	%
Yes, first-hand	516	31.31	444	31.27	72	31.58
Yes, I have witnessed it towards a colleague and/or another user	547	33.19	464	32.68	83	36.40
Yes, both first-hand and witnessed it towards a colleague and/or another user	179	10.86	156	10.99	23	10.09
No, I have not suffered or witnessed it	406	24.64	356	25.07	50	21.93
Overall total	1648	100	1420	100	228	100

**Table 3:** Percentage of aggressions according to their typology.

Type of aggression	Total		Females		Males	
	Cases	%	Cases	%	Cases	%
Verbal	902	54.73	777	54.72	125	54.82
Physical	33	2.00	26	1.83	7	3.07
Both	307	18.63	261	18.38	46	20.18
Not suffered	406	24.64	356	25.07	50	21.93
Overall total	1648	100	1420	100	228	100

**Table 4:** Perpetrators of aggression or violent acts.

Perpetrators	*N*	%
Patients	280	16.99
Relatives	268	16.26
Colleagues	220	13.35
Relatives and patients	244	14.81
Colleagues and patients	66	4.00
Colleagues and relatives	76	4.61
Other users	81	4.91
Not stated	413	25.06
Overall total	1648	100

**Table 5:** Indicators of sexual and workplace harassment at work.

Sexual harassment at work?			Psychological harassment at work?		
	*N*			*N*	%
Yes	126	7.65	Yes	641	38.90
No	1522	92.35	No	1007	61.10
Overall total	1648	100	Overall total	1648	100

**Table 6:** Scores obtained in the engagement dimensions.

In your workplace, have you ever suffered…		Yes	No	Statistical	Effect size
				*Mann–Whitney U*	*Cohen's D*
*Violent act*					
*N* (%)		695 (47.2)	953 (57.8)		
Mean (SD)	Vigour	3.9 (1.4)	4.2 (1.3)	281191.0^*∗∗*^	0.42
	Dedication	4.2 (1.5)	4.5 (1.4)	284235.0^*∗∗*^	0.43
	Absorption	4.1 (1.5)	4.4 (1.4)	291199.0^*∗∗*^	0.44
	UWES-9	4.0 (1.3)	4.4 (1.3)	281847.5^*∗∗*^	0.43

*Threat*					
*N* (%)		679 (41.2)	969 (58.8)		
Mean (SD)	Vigour	3.8 (1.4)	4.2 (1.3)	273493.5^*∗∗*^	0.42
	Dedication	4.2 (1.5)	4.5 (1.4)	276142.0^*∗∗*^	0.42
	Absorption	4.0 (1.5)	4.4 (1.4)	277208.0^*∗∗*^	0.42
	UWES-9	4.0 (1.3)	4.4 (1.3)	271089.5^*∗∗*^	0.41

*Sexual harassment*					
*N* (%)		126 (7.7)	1522 (92.4)		
Mean (SD)	Vigour	3.9 (1.3)	4.1 (1.4)	88725.5	0.46
	Dedication	4.3 (1.4)	4.4 (1.4)	94472.0	0.49
	Absorption	4.3 (1.4)	4.3 (1.4)	92664.5	0.48
	UWES-9	4.2 (1.3)	4.2 (1.3)	93565.5	0.49

*Psychological harassment*					
*N* (%)		641 (38.9)	1007 (61.1)		
Mean (SD)	Vigour	3.7 (1.4)	4.3 (1.3)	239132.5^*∗∗*^	0.37
	Dedication	4.1 (1.5)	4.6 (1.3)	261226.0^*∗∗*^	0.40
	Absorption	4.0 (1.5)	4.4 (1.3)	275839.5^*∗∗*^	0.43
	UWES-9	3.9 (1.4)	4.4 (1.2)	252718.0^*∗∗*^	0.39

^*∗∗*^ *p* < 0.001.

## Data Availability

The data used to support the findings of this study are available from the corresponding author upon reasonable request.
